# Efficacy of Low-Density Lipoprotein Apheresis in an Adult Case of Recurrent Focal Segmental Glomerulosclerosis Post-kidney Transplant

**DOI:** 10.7759/cureus.104388

**Published:** 2026-02-27

**Authors:** Ayda Javanbakht, Kyle Souter, Tomas Armendariz, Christopher B Webb

**Affiliations:** 1 Transfusion Medicine, University of Texas (UT) Southwestern, Dallas, USA

**Keywords:** focal segmental glomerulosclerosis (fsgs), kidney disease, low-density lipoprotein apheresis, low-density lipoprotein cholesterol plasmapheresis, therapeutic plasma exchange (tpe)

## Abstract

Recurrence of focal segmental glomerulosclerosis (FSGS) in the transplanted kidney has been reported in renal transplant cases. The management often involves therapeutic plasma exchange (TPE) and immunomodulatory therapies with a variable response. In patients with FSGS, dyslipidemia can contribute to irreversible renal injury. We present a 26-year-old man with recurrent FSGS in his second transplanted kidney which was controlled partially with aggressive TPE for over 3.5 years. He achieved complete remission following the completion of the low-density lipoprotein apheresis (LDL-A) protocol which consists of two procedures per week for three consecutive weeks, followed by one procedure per week for the 12 consecutive weeks (total of 18 procedures). He has been in remission for over four years after a single course of LDL-A. This report supports the use of LDL-A for recurrent FSGS post-kidney transplant in adults.

## Introduction

Focal segmental glomerulosclerosis (FSGS) is a clinicopathologic entity characterized by podocyte injury and is the leading glomerular cause of end-stage renal disease (ESRD) in the United States [1]. The chance of FSGS in a transplanted kidney is approximately 30-60%, with higher rates up to 80-100% on second or subsequent grafts if the first was lost to recurrence. Management includes immunosuppression with high-dose corticosteroids and/or calcineurin inhibitors (CNIs), though a significant percentage of patients remain resistant to treatment. Therapeutic plasma exchange (TPE) has been reported as an adjunct treatment in primary FSGS since the 1980s [2]. It remains an American Society for Apheresis (ASFA) Category I indication for recurrent FSGS after renal transplantation [3], by removing the putative circulating permeability factor that plays a role in glomerular injury.

Patients with FSGS have dyslipidemia, and low-density lipoprotein apheresis (LDL-A) could help remove dysfunctional lipoproteins, lipids, and inflammatory factors that cause glomerular scarring and damage. LDL-A (Kaneka Corporation, Osaka, Japan) has been described as a therapeutic option for medication-refractory or recurrent FSGS, with the aim of achieving complete or partial remission [4]. In 2018, LDL-A was approved by the Food and Drug Administration (FDA) under a humanitarian device exemption (HDE) as a potential therapeutic option for recurrent or medication-refractory FSGS in adults (ASFA Category II). We are reporting an adult patient with recurrent FSGS post-renal transplant in remission for over four years following LDL-A therapy.

## Case presentation

A 26-year-old man was first diagnosed with nephrotic syndrome (NS) in 2007 during evaluation for periorbital edema and respiratory distress related to pleural effusion. The patient was diagnosed with FSGS through a renal biopsy. He failed high-dose corticosteroids and cyclosporine (CsA), requiring peritoneal dialysis in 2008. In 2012, he underwent the first renal transplant, but the graft failed despite all treatments. He received the second renal transplant in 2016 with a biopsy-proven recurrence of FSGS within days of transplant. He underwent an acute course of TPE followed by twice-weekly treatments. He transferred his care for the TPEs to our Apheresis unit in 2017. He continued weekly to twice-weekly TPEs (1.0 plasma volume, using 5% albumin as replacement fluid), completing a total of 174 TPE procedures over 3.5 years with partial remission. Table 1 demonstrates relevant labs during TPEs. Renal status was monitored with a biweekly total protein (TP)/creatinine (Cr) ratio, and the patient was treated with CsA, mycophenolic acid, prednisone, and monthly infusions of intravenous immunoglobulin (IVIG) and ofatumumab.

**Table 1:** Relevant labs over the treatment course eGFR: estimated glomerular filtration rate; BUN: blood urea nitrogen; Cr: creatinine; TP/Cr: total protein-to-creatinine ratio; TPE: therapeutic plasma exchange; LDL-A: low-density lipoprotein apheresis

Date	eGFR (mL/min/1.73 m^2^)	BUN (mg/dL)	Cr (mg/dL)	BUN/Cr	Urine protein (mg/dL)	Urine Cr (mg/dL)	TP/Cr	Notes
9/20/2017	51	27	1.7	15.9	513	103.8	4.9	TPE #1
9/17/2018	55	25	1.6	16	750	85.8	8.7	TPE year 2
9/24/2019	58	27	1.6	17.3	600	134.3	4.5	TPE year 3
9/23/2020	>60	30	1.3	23.1	319	58.4	5.5	TPE year 4
5/13/2021	>60	32	1.3	24.1	493	36.1	13.7	TPE #194 (final)
5/19/2021	52	29	1.7	17.1	436	79.3	5.5	LDL-A #1
6/1/2021	45	39	1.9	20.2	464	69.8	6.6	LDL-A #5
6/29/2021	57	28	1.6	17.7	404	64.4	6.3	LDL-A #10
7/21/2021	56	36	1.6	22.5	304	39.9	7.6	LDL-A #13; steroid taper began
7/28/2021	>60	48	1.4	35.6	282	38	7.4	LDL-A #14
8/4/2021	59	46	1.5	30.1	206	38.6	5.3	LDL-A #15
8/11/2021	60	46	1.5	30.5	185	49.8	3.7	LDL-A #16
8/16/2021	51	55	1.7	32	199	64.2	3.1	LDL-A #17
8/25/2021	57	43	1.6	27.2	181	62	2.9	After LDL-A #18 (final)
5/13/2025	43	51	2.1	24	124	70	1.8	4 years post-LDL-A
11/26/2025	43	60	2.1	29	160	43	3.7	Most recent labs

**Figure 1:**
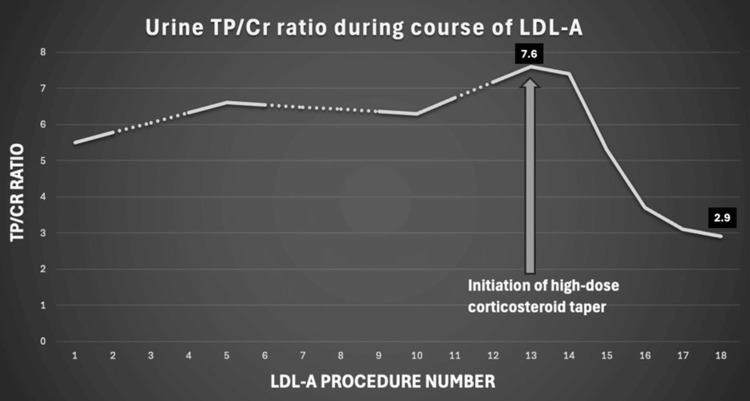
TP/Cr ratio during LDL-A treatment course LDL-A: low-density lipoprotein apheresis; TP/Cr: total protein-to-creatinine ratio

In May 2021, he transitioned to LDL-A (upon FDA approval under HDE for this indication), beginning with two procedures per week for three consecutive weeks, followed by one procedure per week for the 12 consecutive weeks (total of 18 procedures). LDL-A was performed with the LIPOSORBER® LA-15 System (Kaneka Medical America, LLC, New York, New York, United States), and 1.5 plasma volume was processed with unfractionated heparin as an anticoagulant. All procedures were tolerated well with no adverse events. Before TPE #13, he was started on a prednisone taper (60 mg daily, decreased by 10 mg every week). Prednisone was added to improve the LDL-A efficacy. No other medication changes were made during LDL-A other than discontinuing IVIG. Over the next five weeks, TP/Cr steadily decreased from 7.6 to 2.9. His dyslipidemia has improved. He has since remained symptom-free, with labs remaining within acceptable ranges. The patient has returned to normal life without the need for further apheresis or dialysis, and his fistula was removed in 2025 with no new biopsy. He continues to be on prednisone 5 mg twice a day, Myfortic 360 mg twice a day, and CsA 125 mg in the morning and 100 mg at night with stable labs (Figure 1).

## Discussion

We report a remission of recurrent FSGS with LDL-A following the second renal transplantation in an adult who was TPE dependent for 3.5 years with several immunosuppression therapies. His graft function has been maintained for over four years post-LDL-A. The patient is still on immunosuppression but has stable renal function. Future studies are warranted to exclude the confounding effects of immunosuppressive therapy.

The recurrent idiopathic FSGS after renal transplant can lead to rapid allograft failure, and not all patients respond to immunosuppressive therapy. In early 1985, Zimmerman [5] reported the use of TPE in a recurrent FSGS case after renal transplant, in which TPE reduced proteinuria and stabilized renal function. Post-transplant recurrence remains a Category I indication for TPE; however, clinical response is variable.

LDL-A is a selective, membrane filtration apheresis technique that selectively removes apolipoprotein B100-containing lipoproteins from plasma via two replenishable adsorption columns. Evidence is limited to small, nonrandomized studies in heterogeneous NS populations. These studies showed a response rate ranging from 54% to 86% mostly in the pediatric population, with response generally defined as a partial or complete remission [6,7]. Hattori et al. [8] combined 12 LDL-A sessions with the addition of high-dose steroids in procedure #7 in a cohort of 11 pediatric patients with biopsy-proven FSGS who were steroid-resistant and had failed CsA. A total response rate of 76% was observed, with 5 achieving "complete remission" (defined as proteinuria <4 mg/m^2^/hr for three days, with normalization of serum albumin and cholesterol with stable renal function). These studies paved the way for FDA approval for pediatric patients in 2013 and approval under HDE for adult patients in 2018.

The therapeutic effects of LDL-A likely involve multiple mechanisms. Chronic dyslipidemia in NS contributes to a "lipid nephrotoxicity", which has deleterious effects on the glomerulus, podocytes, as well as the tubulointerstitium [9]. LDL-A may lower levels of potentially harmful complement proteins and lipoprotein-bound coagulation factors and may remove the circulating permeability factor. Improvement in steroid-responsiveness after LDL-A is a notable observation from several studies [7], and future studies are needed to evaluate the confounding effects of immunosuppressive therapy. The patient had the greatest benefit following the initiation of high-dose corticosteroids at the time of procedure #13, with rapid weekly improvement in TP/Cr ratio during the remainder of his treatment course. Regulating dyslipidemia may reduce nephrotoxicity and enhance corticosteroid and CNI responsiveness.

LDL-A typically involves a more limited course of 12-18 weeks, in contrast to long-term TPEs, with many patients achieving and maintaining remissions for years following treatment. The POLARIS study, the largest prospective study on the long-term effects of LDL-A on drug-resistant NS, enrolled 58 refractory NS adult patients with an average age of 55.8±18.1 from 40 centers, 44 of whom were followed for two years. Of those, 47.7% achieved and remained in remission during that time [10]. Improvement of labs at the end of the treatment course was associated with favorable outcomes. Despite years of treatment with TPE, our case achieved significant improvement in proteinuria after LDL-A and has remained in remission for over four years.

The limitation of this single case report is the lack of a control group.

## Conclusions

Improvement of kidney function-related labs and decreasing the need for dialysis at the end of the LDL-A treatment course were associated with favorable outcomes in FSGS disease. Despite years of treatment with TPE, our FSGS case achieved significant improvement in proteinuria after LDL-A and has remained in remission for over four years. The successful treatment outcome of our case supports that LDL-A might be considered in patients with recurrent FSGS post-transplant, particularly if the TPE has failed.

## References

[1] Rosenberg AZ, Kopp JB (2017). Focal segmental glomerulosclerosis. Clin J Am Soc Nephrol.

[2] Shariatmadar S, Noto TA (2002). Therapeutic plasma exchange in recurrent focal segmental glomerulosclerosis following transplantation. J Clin Apher.

[3] Connelly-Smith L, Alquist CR, Aqui NA (2023). Guidelines on the use of therapeutic apheresis in clinical practice - evidence-based approach from the Writing Committee of the American Society for Apheresis: the Ninth Special Issue. J Clin Apher.

[4] Shah L, Hooper DK, Okamura D (2019). LDL-apheresis-induced remission of focal segmental glomerulosclerosis recurrence in pediatric renal transplant recipients. Pediatr Nephrol.

[5] Zimmerman SW (1985). Plasmapheresis and dipyridamole for recurrent focal glomerular sclerosis. Nephron.

[6] Muso E, Mune M, Hirano T (2015). A prospective observational survey on the long-term effect of LDL apheresis on drug-resistant nephrotic syndrome. Nephron Extra.

[7] Muso E, Mune M, Fujii Y (2001). Significantly rapid relief from steroid-resistant nephrotic syndrome by LDL apheresis compared with steroid monotherapy. Nephron.

[8] Hattori M, Chikamoto H, Akioka Y (2003). A combined low-density lipoprotein apheresis and prednisone therapy for steroid-resistant primary focal segmental glomerulosclerosis in children. Am J Kidney Dis.

[9] Agrawal S, Zaritsky JJ, Fornoni A, Smoyer WE (2018). Dyslipidaemia in nephrotic syndrome: mechanisms and treatment. Nat Rev Nephrol.

[10] Muso E, Sakai S, Ogura Y (2022). Favorable therapeutic efficacy of low-density lipoprotein apheresis for nephrotic syndrome with impaired renal function. Ther Apher Dial.

